# Supplementary material for: Enabling Automatic Generation of Protein–Ligand Complex Data Sets with Atomistic Detail
Source: J Chem Inf Model. 2026 May 8;66(10):5824–37. doi: 10.1021/acs.jcim.6c00150 (PMC13213827; doi:10.1021/acs.jcim.6c00150)
Supplement: Supplementary file 1 [file ci6c00150_si_001.pdf]

Supporting Information: Enabling automatic generation of protein-ligand complex datasets with atomistic detail

Torben Gutermuth,[†] Emanuel S. R. Ehmki,[†,‡] Florian Flachsenberg,[†,¶] Patrick Penner,[†,§] Sophia M. N. Hönig,[†,¶] Tobias Harren,[†] and Matthias Rarey[*,†]

† *University of Hamburg, ZBH - Center for Bioinformatics, Albert Einstein Ring 8-10, 22761 Hamburg, Germany*

‡*Current Address: Pfizer Pharma GmbH, Friedrichstraße 110, 10117 Berlin*

¶*Current Address: BioSolveIT GmbH, An der Ziegelei 79, 53757 Sankt Augustin, Germany*

§*Current Address: Global Discovery Chemistry, Novartis Biomedical Research, Basel, Switzerland*

E-mail: matthias.rarey@uni-hamburg.de

1 StrAcTable Documentation

An exact documentation on each column in the StrAcTable and therefore each column in the respective tables can be found in machine-readable json format in the supporting Information. A list of particularly important columns is given in Table S1.

Table S1: List of particularly important columns and their explanations. For a full list for all columns please see the supporting information.

Column	Explanation
pdb	PDB id
name	NAOMI name, unique per PDB entry
skip_reason	Skip reason
usmiles	usmiles of the compound
activity.standard_value	Value of activity
activity.standard_relation	Relation of activity (e.g. $=,>$)
activity.standard_type	Type of activity (e.g. IC50, EC50)
activity.standard_units	Unit of activity (always nM for IC50,KI,KD,...)
assay.chembl_id	ChEMBL assay id
chembl_ligand.chembl_id	ChEMBL ligand id
target.chembl_id	ChEMBL target id
Target_Type	Target Type (e.g. single protein, protein familiy)
chembl_activity_data_validity_comment	Data validity comment in the ChEMBL
chembl_activity_potential_duplicate	ChEMBL potential duplicate flag
chembl_assay_assay_type	ChEMBL assay type (e.g. B for binding)
chembl_assay_confidence_score	ChEMBL assay confidence score (9 best)
Level_pm	Protein matching level
small_molecule_matching_confidence_level.comment	Molecule matching level
N_mutations	Number of mutations in active site
mw	Weight of the molecule $[\frac{g}{mol}]$
resolution	Resolution of the PDB structure
OWAB	Occupancy of weighted atomic b-factors[S1]
$EDIA_m$	$EDIA_m$[S2]
ligand_structure	Merge indicator for ligand and structure data (left_only, right_only, both)
activities_structureligand	Merge indicator for activity and ligand/structure data

2 Further StrAcTable analysis

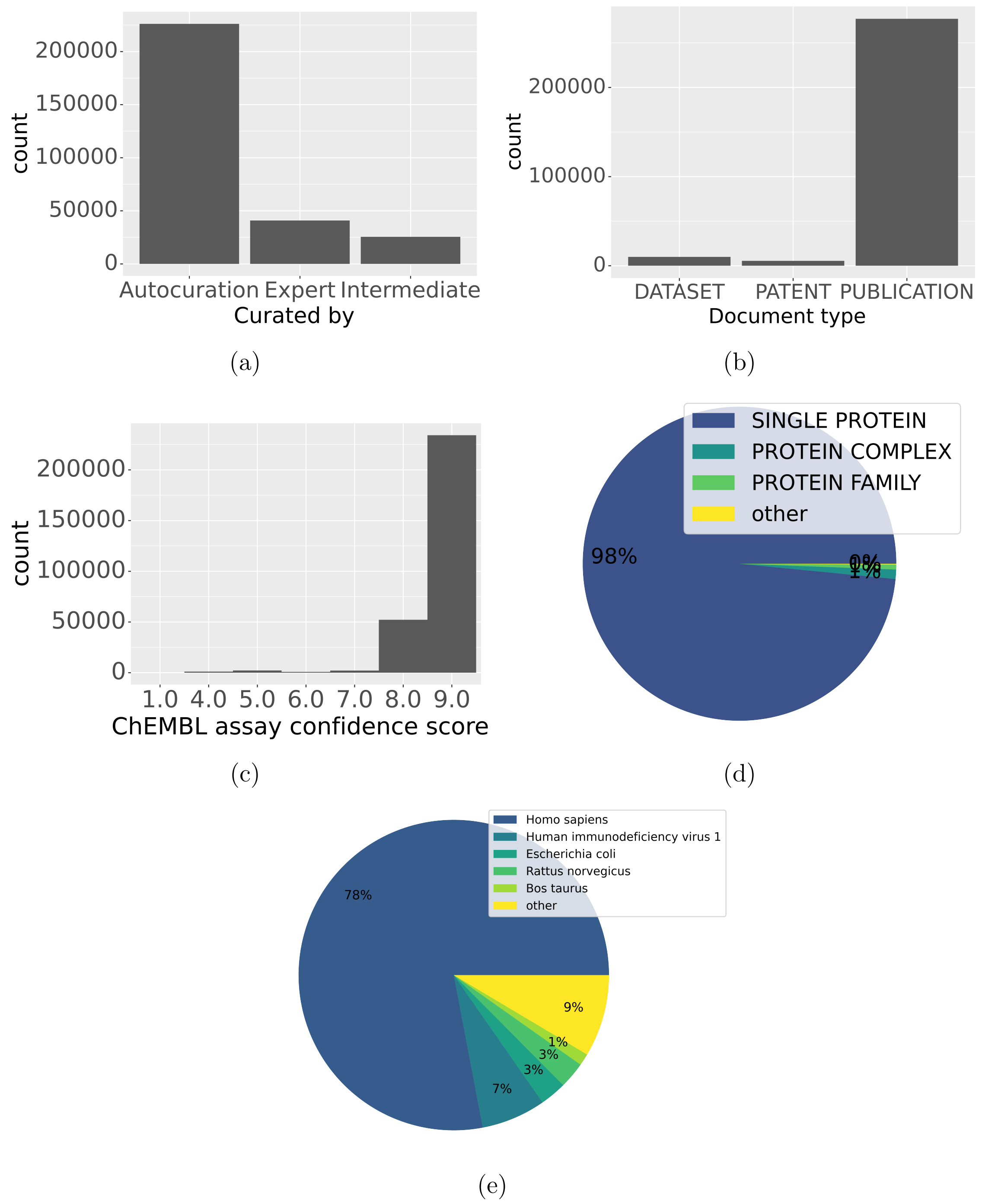

Figure S1: Analysis of the occurences of different curation strategies (a), document types (b), assay confidence scores (c), target types (d) and organisms (e) for all entries in StrAcTableF containing data from all respective tools.

3 LigandExtractor skip reasons

The LigandExtractor tool was developed to list all potential ligands in a PDB file and report any problems with a ligand that either arise from inconsistencies with the annotated metadata within the PDB file or from its interpretation using the NAOMI cheminformatics library.[S3,S4] It was first described in Flachsenberg et al. (2024).[S5] Table S6 of Flachsenberg et al. (2024)[S5] only contained a simple list of the occurring problems (i.e., skip reasons) along with their frequency. Here, we provide, for each possible ligand classification, at least one exemplary PDB code for further illustration. All ligands and residues are identified by the tuple `<resName>_<chainID>_<resSeq><iCode>`.

Note that the metadata within PDB files might be updated at any time in the future. Therefore, we specify for each PDB code the most recent version and revision date where the described behavior is known to occur.

NotSkipped

No problem was detected. An example is ligand `SOX_B_1559` in *1gm8*[S6,S7] (v1.4, 2024-05-08).

LigandIsMetal

The ligand is an isolated metal ion. One example is the Ca^{2+} ion `CA_B_1558` in *1gm8*[S6,S7] (v1.4, 2024-05-08).

LigandIsCovalentOrHeteroResidue

The (potential) ligand is treated as being part of a macromolecule. This includes non-standard residues in protein chains (e.g., phosphorylated serine `SEP_A_46` in *1fu0*[S8,S9] (v1.4, 2024-10-16), and covalent ligands (e.g., ligand `FHR_A_405` in *6lze*[S10,S11] (v2.6, 2024-10-23)).

LigandIsIncomplete

In REMARK 470[S13] or REMARK 610,[S14] the ligand is annotated to have missing heavy

atoms. One example is ligand P33_A_201 in *4xjy*[S15] (v2.1, 2024-11-06).

LigandContainsMetal

The ligand molecule contains a metal atom. A common example is the iron-containing prosthetic group heme, e.g., ligand HEM_A_143 in *1g9v*[S16,S17] (v1.4, 2023-08-09).

LigandIsPolymerWithMissingCoordinates

In general, oligopeptides and oligonucleotides with up to nine residues are considered to be ligand molecules. However, we check standard amino acids and nucleic acids for completeness, i.e., if any expected atom is missing. Here, either a side chain atom can be missing (e.g., ligand ARG_P_194_ARG_P_195_ARG_P_196_GLN_P_197_TPO_P_198 in *7ort*[S18] (v1.2, 2024-11-06) is missing side chain atoms in the N-terminal arginine) or an oxygen of the C-terminal carboxy group (e.g., ligand GLY_B_1_ALA_B_2_LYS_B_3 in *1gdn*[S19,S20] (v1.4, 2024-11-06)). The latter case also includes covalent peptide ligands, e.g., ligand VAL_A_4_GLU_A_5_PRO_A_6_ILE_A_7 in *1h9l*[S21,S22] (v1.4, 2024-10-16), where an acyl–enzyme complex is formed.

LigandWasSkippedByAltloc

This is connected to the handling of alternate locations: Here, we globally use the first annotated alternate location identifier (usually 'A') for all residues. However, if a residue is exclusively annotated in another alternate location, it will not be built. An example is ligand QTO_A_201 in *5sqm*[S23,S24] (v1.2, 2023-09-20), where the residue QTO_A_201 is exclusively annotated with alternate location 'B'.

LigandIsUnknownMetal

We found a metal ion that is not given in the HET section.[S25] This can happen if there are metal-containing non-standard amino acid residues (e.g., ligand BFD_A_170 (aspartate beryllium trifluoride) in *4xq0*[S26,S27] (v1.6, 2024-11-13) in the protein. Here, the metal is

treated as a separate entity (BE_A_170) that is not explicitly listed in the HET section.

A similar effect can also happen for heme prosthetic groups that are covalently bound to the protein (e.g., metal FE_A_201 in *4xxl*[S28,S29] (v2.1, 2024-10-16) that is actually part of the ligand HEC_A_201).

LigandIsIncompleteNaomi

The ligand molecule could not be correctly built by our NAOMI library,[S3,S4] and the molecule is decomposed into multiple parts. One example is ligand BFD_A_170 in *4xq0*[S26,S27] (v1.6, 2024-11-13) where the BFD (aspartate beryllium trifluoride) is decomposed into the aspartate residue (part of the protein), the beryllium ion (see also 'LigandIsUnknownMetal') as well as three fluoride ions. Of the latter, only one is part of the complex.

LigandCouldNotBeBuilt

These molecules cannot be built by our NAOMI library[S3,S4] at all. This includes molecules with unsupported valences, e.g., bonding in carborane 34B_A_1188 in *2c2s*[S30,S31] (v1.5, 2024-05-01).

Furthermore, it also includes metal ions for which the charge could not be determined from the ATOM[S32] or HETATM[S33] section alone. One example is TL_A_114 in *2hbn*[S34,S35] (v1.4, 2023-08-30) which could generally be Tl^{+} and Tl^{3+}. (This ambiguity could be resolved by considering the HET code that designates the ion as Tl^{+}.)

LigandIsUnknownAtom

The ligand could not be built by the NAOMI library[S3,S4] and has the HET code UNX, UNL, or UNK. Here, the interpretation usually fails because the ligand only consists of an unknown atom (e.g., ligand UNX_A_401 in *4xx6*[S36] (v2.2, 2024-10-23)).

LigandHasUnknownHetcode

The ligand is not a standard amino acid/nucleic acid, and it is not listed in the FORMUL

section,[S37] or the given formula for the ligand is empty. An example is ligand `XWD_R_401` in *8x3l*[S38,S39] (v1.2, 2025-06-25) where the formula is empty.

LigandIsPolymerWithChainBreak

In general, oligopeptides and oligonucleotides with up to nine residues are considered to be ligand molecules. However, we require the oligomeric ligand to have a different chain name than the macromolecule or other oligomeric ligands (if it has the same type, i.e., nucleic acid/amino acid). This is done to ensure that there are no missing bonds, no missing residues, and that a fragment of a macromolecule is not incidentally interpreted as a ligand. An example of a chain break due to a missing bond are the two (pseudo)ligands `CYS_D_1_ASP_D_2` and `ALA_D_4_ASN_D_5_PHE_D_6_LYS_D_7` in *1p13*[S40,S41] (v1.3, 2024-12-25). These should actually form a heptapeptide with an *O*-Phosphotyrosine in between (`PTR_D_3`). However, the distances between `PTR_D_3` and `ASP_D_2`, as well as `ALA_D_4`, are both too large to actually be recognized as peptide bonds.

Similarly, the two (pseudo)ligands `LEU_J_77_SER_J_78` and `GLY_J_84_VAL_J_85` in *6gjh*[S42,S43] (v1.3, 2024-10-16) should actually form a longer peptide. Here, not only bonds, but also residues in between are missing. The latter is confirmed in the corresponding primary publication.[S43]

LigandIsCovalentOrIncompletePolymer

There is a connection given in the LINK section,[S44] but the residues are not attached to each other. This can, for example, be a non-detected covalent ligand, e.g., `8WY_B_1102` in *5v4q*[S45,S46] (v1.5, 2023-10-04) that should be connected to `THR_B_381` via two bonds, but the connection was not correctly interpreted by the NAOMI library.[S3,S4]

An alternative reason is an annotated LINK between a ligand and a metal ion that is never modelled by the NAOMI library,[S3,S4] e.g., ligand `COM_A_1205` in *7b2c*[S47,S48] (v1.1, 2024-01-31) is annotated to be bound to the nickel within cofactor `USN_A_1201`.

LigandIsPolymerWithDifferentChains

In general, oligopeptides and oligonucleotides with up to nine residues are considered to be ligand molecules. However, we check if all monomers come from the same chain. If this is not the case, we do not consider this to be a ligand. The rationale behind this filter is that usually oligomers should be defined as their own chain (see also 'LigandIsPolymerWithChainBreak').

On the one hand, this prevents molecules from being connected that obviously should not be connected: Analyzing the connection of ligands `U10_L_303` and `LDA_M_404` in *8vtj*[S49,S50] (v1.1, 2025-09-24) show unreasonable bond angles of 148 and 135 degrees at sp^3-carbons.

On the other hand, the assumption of homogeneous chain IDs for oligomeric ligands is not always true, especially when oligosaccharides are involved: Here, often one/the sugar part has its own chain ID, e.g., xylotriose `XYP_D_1_XYP_D_2_XYP_A_1001` in *4xuq*[S51,S52] (v2.1, 2024-01-10) or the glycosylated serine `NGA_E_1_GAL_E_2_SER_A_147` in *1y2x*[S53,S54] (v2.2, 2024-04-03).

LigandHasUnexpectedFormula

For a monomeric ligand, the molecular formula excluding hydrogens calculated from the molecule read by NAOMI[S3,S4] differs from the one specified in the FORMUL section.[S55] One example is ligand `4US_A_701` in *5bq0*[S56,S57] (v1.1, 2024-03-06), where in the considered alternate location 'A', the nitrogen atom 'N4' is missing, leading to a different molecular formula.

InternalError

Other, non-expected problems were detected. The only known case is ligand `TPO_A_158X` (and `TPO_B_158X`) in *8haw*[S58,S59] (v1.1, 2024-10-23). The problem here is an inconsistent insertion code, i.e., in the HET section,[S60] the residue is announced as `TPO_A_158X` (with upper case insertion code), but in the coordinate section, it is labeled as `TPO_A_158x` (with lower case insertion code).

LigandIsMissingInModel1

Table S6 in Flachsenberg et al. (2024)[S5] counted exactly one case where the PDB file contained multiple models, but the ligand in question was not part of the first model. This referred to ligand `U5P_B_903` in *2icy*.[S61,S62] However, the metadata has been corrected for the current entry version 1.4 (2023-08-30). Therefore, currently, no example for this ligand classification exists anymore.

4 Usage of StrAcTable

As the StrAcTable is already being used in our lab to develop further benchmarks and analysis, we want to highight some common pitfalls or usage advice to our users.

4.1 Unique Keys in the StrAcTable

When working with the StrAcTable, there are many approaches which require a unique key for a protein-ligand pair. For all PDB structures, the combination of PDB-ID and Naomi ligand name (which is a combination of the ligand HETcode, chain and residue ID) describes a unique protein-ligand pair. When also taking activities into consideration, the combination of the aforementioned key with the activity id should be unique. The only exception to this rule are molecules which cannot be read correctly by Naomi like inorganic clusters. If inorganic clusters are found in the PDB (one example is the ligand JGH in the PDB ID 6qsh) they are read into multiple different molecule datastructures using NAOMI and each part of the complete molecule is handled separately for calculations like EDIAm. Thankfully, these cases as well as many other in which NAOMI either struggles to correctly model the experimental reality or the experimental model is faulty can be filtered out using the Skipreasons computed using the LigandExtractor.

4.2 Handling measurements of racemic mixtures

While ligands modelled in crystal structures have to be modelled as one specific isomer, it is quite frequent to use racemic mixtures when measuring binding affinities (see Figure S2). To avoid using datapoints that have been measured using racemic mixtures, users should only use the two best ligand matching criteria (Full inchi match/ chiral usmiles match), as in these cases the identical isomer is mandatory.

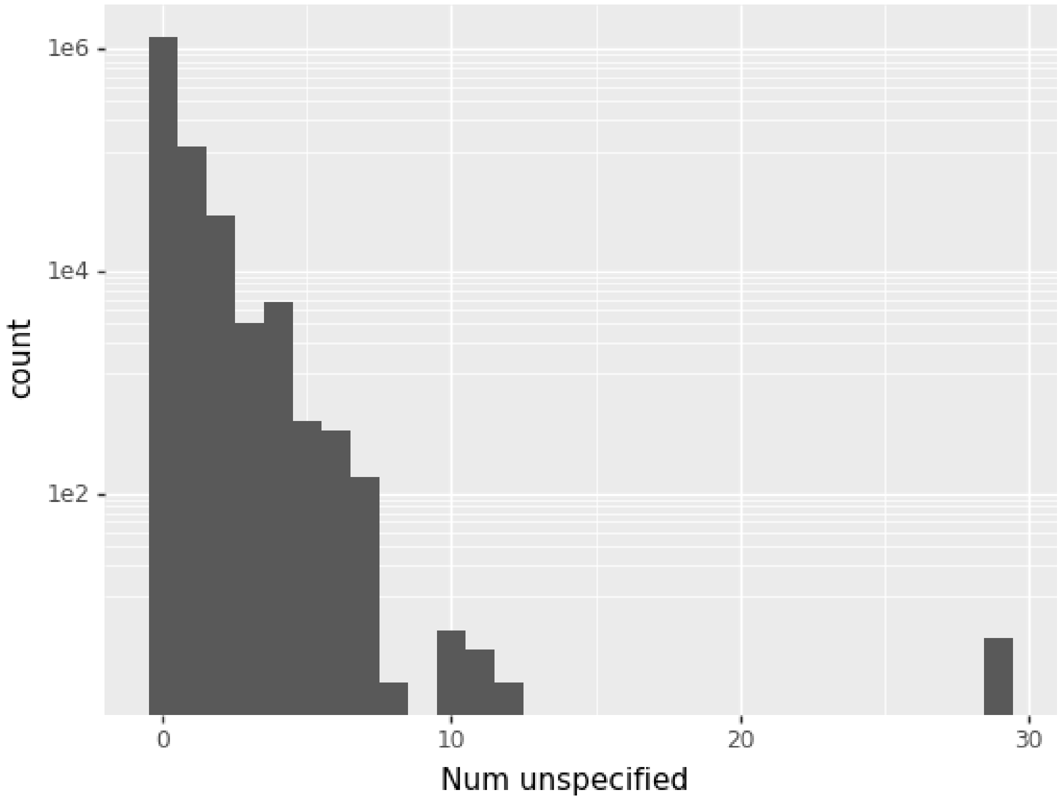

Figure S2: Number of unspecified stereocenters according to RDKit[S63] for all entries in the filtered StrAcTable that are not skipped and have activity data.

5 Changes in supplied electron densities

In August 2022, we learned that the electron densities supplied through EMBL-EBI had changed. In some structures, the electron density now does not encompass the complete modeled structure (e.g., PDB-ID `7AQI`[S64,S65]), or the electron density is moved in three-dimensional space compared to the modeled structure (e.g., PDB-ID `5RT2`[S66,S67]). While this can be fixed by extending and moving the electron density, this step is not initially done in the StructureProfiler. To circumvent this problem, we used GEMMI[S68] to extend and use the extended electron densities. However, for some structures (e.g., PDB-ID `1MZC`[S69,S70] or `4OCO`[S71,S72]), this results in electron densities that are not readable using the StructureProfiler due to problems of GEMMI[S68] with the dsn6 electron density format. To achieve a solution that is generalizable for all structures in the PDB, we generate the electron density from the mtz file encompassing the complete structural model using GEMMI,[S68] circumventing any issues with specific electron density file formats. However, in 2024 the mtz files were discontinued, which necessitated another change to generate the electron densities from the 2fo-fc cif files. Both approaches have been validated against the old $EDIA_m$ values before 2022. This workflow has been added to ProteinsPlus.[S73–S75]

6 Protein-ligand matching

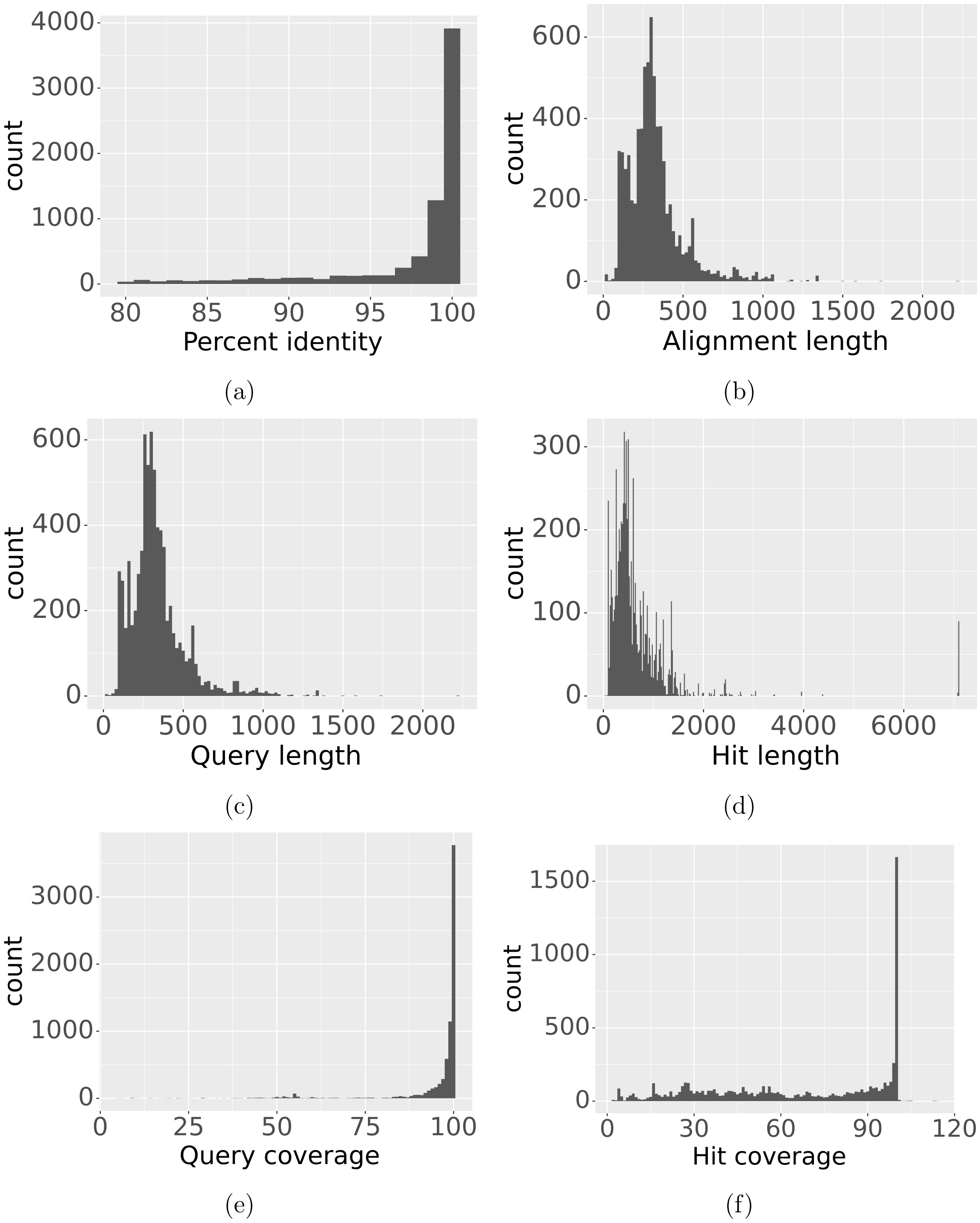

Figure S3: Histograms of the percent identity (a), alignment length (b), query length (c), hit length (d), query coverage (e), and hit coverage (f) for all unique blast match ids in the raw StrAcTable with at least one matching entry with structure and ligand data.

6.1 Validation of the protein matching heuristic

ActivityFinder provides users with many possible matches to ChEMBL targets and activities for a single protein-ligand pair or PDB structure, which poses the challenge of finding the best-matching ChEMBL target for each in an automated manner. Examples of multiple ChEMBL targets for a given entry include targets from different organisms, slightly different versions of the same protein, or other target types such as single proteins, protein complexes, or protein families. The PDB structures and targets of the DUD-E[S76] dataset were used to develop and test a heuristic algorithm for automatic target selection. To compare the ability of a knowledgeable user and ActivityFinder with our heuristic to retrieve the most relevant ChEMBL target, a manual matching was conducted using metadata in the PDB and ChEMBL for each of the 102 targets and their respective structures (see Table S2). In the activity mode, no matching activities were found in 23 cases; in the target mode, two entries (Target AOFB and CASP3) produced no output, resulting in 100 possible targets in the target mode and 79 in the activity mode. In addition, for the activity mode, there are three PDBs with multiple ligands where activities can be found, meaning there are 82 total entries. Focusing on the activity mode, 73 of 82 entries (89.02%) can be correctly linked. The nine missed entries were due to activity data being measured against a slightly different target, for example, the protein of a different organism (e.g., DAY/`3BQD`[S77,S78]) or a protein complex rather than a single protein (e.g., GRIA2, `3KGC`[S79]). In each case, the manual ChEMBL target was not linked by ActivityFinder, so the heuristic could not select it. In target mode, the manually assigned target is reproduced in 96 of 100 potential cases (96.00 %). In one case, the heuristic selects a specific subunit of the protein rather than the entire complex (FNTA, `3E37`[S80,S81]). Twice (AKT1 / `3CQW`[S82,S83] and HIVPR / `1XL2`[S84,S85]), the mismatch is a failure in our heuristic, as the manual ChEMBL target and a very similar target can only be differentiated by the tiebreaker. In the other case (HIVINT / `3NF7`[S86,S87]) , the found ChEMBL target has a slightly better sequence match. Since StrAcTable will be

provided both unfiltered and filtered to the best-matching ChEMBL target, users can use our validated matching or create their own algorithm.

Table S2: Detailed results of the protein matching experiment between manually matched ChEMBL targets based on metadata and the results of the ActivityFinder. Target Column describes the manually matched target, Found target describes the ActivityFinder target if they are different. Reason describes the reason for which a different target was found and Difference describes the difference between the two targets. Mode describes the mode of the ActivityFinder, either Activity mode only finding targets with ligands with matching activities or target mode ignoring data of present ligands.

Name	PDB	Target	Found target	Reason	Difference	Type
AA2AR	3eml	CHEMBL251				Activities
AA2AR	3eml	CHEMBL251				Targets
ABL1	2hzi	CHEMBL1862		No Activities found		Activities
ABL1	2hzi	CHEMBL1862				Targets
ACE	3bkl	CHEMBL1808		No Activities found		Activities
ACE	3bkl	CHEMBL1808				Targets
ACES	1e66	CHEMBL4780				Activities
ACES	1e66	CHEMBL4780				Targets
ADA	2e1w	CHEMBL2966	CHEMBL1910	Molecule not measured against perfect target	Organism difference	Activities
ADA	2e1w	CHEMBL2966				Targets
ADA17	2oi0	CHEMBL3706				Activities
ADA17	2oi0	CHEMBL3706				Targets
ADRB1	2vt4	CHEMBL213		No Activities found		Activities
ADRB1	2vt4	CHEMBL213				Targets
ADRB2	3ny8	CHEMBL210				Activities
ADRB2	3ny8	CHEMBL210				Targets
AKT1	3cqw	CHEMBL4282				Activities
AKT1	3cqw	CHEMBL4282	CHEMBL262	Target number is smaller	Different target	Targets
AKT2	3d0e	CHEMBL2431				Activities
AKT2	3d0e	CHEMBL2431				Targets
ALDR	2hv5	CHEMBL1900				Activities
ALDR	2hv5	CHEMBL1900				Targets
AMPC	1l2s	CHEMBL2026				Activities
AMPC	1l2s	CHEMBL2026				Targets
ANDR	2am9	CHEMBL1871		No Activities found		Activities
ANDR	2am9	CHEMBL1871				Targets
AOFB	1s3b	CHEMBL2039		No Activities found		Activities
AOFB	1s3b	CHEMBL2039		No valid ligands found		Targets
BACE1	3l5d	CHEMBL4822				Activities
BACE1	3l5d	CHEMBL4822				Targets
BRAF	3d4q	CHEMBL5145				Activities
BRAF	3d4q	CHEMBL5145				Targets

CAH2	1bcd	CHEMBL205		Activities
CAH2	1bcd	CHEMBL205		Targets
CASP3	2cnk	Nothing found	No Activities found	Activities
CASP3	2cnk	Nothing found		Targets
CDK2	1h00	CHEMBL301		Activities
CDK2	1h00	CHEMBL301		Targets
COMT	3bwm	CHEMBL2023	No activities found	Activities
COMT	3bwm	CHEMBL2023		Targets
CP2C9	1r9o	CHEMBL3397		Activities
CP2C9	1r9o	CHEMBL3397		Targets
CP3A4	3nxu	CHEMBL340		Activities
CP3A4	3nxu	CHEMBL340		Targets
CSF1R	3krj	CHEMBL1844		Activities
CSF1R	3krj	CHEMBL1844		Targets
CXCR4	3odu	CHEMBL2107		Activities
CXCR4	3odu	CHEMBL2107		Targets
DEF	1lru	CHEMBL1795101		Activities
DEF	1lru	CHEMBL1795101		Targets
DHI1	3frj	CHEMBL4235		Activities
DHI1	3frj	CHEMBL4235		Targets
DPP4	2i78	CHEMBL284	No Activities found	Activities
DPP4	2i78	CHEMBL284		Targets
DRD3	3pbl	CHEMBL234		Activities
DRD3	3pbl	CHEMBL234		Targets
DYR	3nxo	CHEMBL202		Activities
DYR	3nxo	CHEMBL202		Targets
EGFR	2rgp	CHEMBL203		Activities
EGFR	2rgp	CHEMBL203		Targets
ESR1	1sj0	CHEMBL206		Activities
ESR1	1sj0	CHEMBL206		Targets
ESR2	2fsz	CHEMBL242		Activities
ESR2	2fsz	CHEMBL242		Targets
FA10	3kl6	CHEMBL244		Activities
FA10	3kl6	CHEMBL244		Targets
FA7	1w7x	CHEMBL3991		Activities
FA7	1w7x	CHEMBL3991		Targets
FABP4	2nnq	CHEMBL2083		Activities
FABP4	2nnq	CHEMBL2083		Targets
FAK1	3bz3	CHEMBL2695		Activities
FAK1	3bz3	CHEMBL2695		Targets

FGFR1	3c4f	CHEMBL3650				Activities
FGFR1	3c4f	CHEMBL3650				Targets
FKB1A	1j4h	CHEMBL1902		No Activities found		Activities
FKB1A	1j4h	CHEMBL1902				Targets
FNTA	3e37	CHEMBL2094108		No Activities found		Activities
FNTA	3e37	CHEMBL2094108	CHEMBL271	Target type	Subunit of whole protein	Targets
FPPS	1zw5	CHEMBL1782				Activities
FPPS	1zw5	CHEMBL1782				Targets
GCR	3bqd	CHEMBL2034	CHEMBL3368	Molecule not measured against perfect target	Organism difference	Activities
GCR	3bqd	CHEMBL2034				Targets
GLCM	2v3f	CHEMBL2179		No Activities found		Activities
GLCM	2v3f	CHEMBL2179				Targets
GRIA2	3kgc	CHEMBL3503	CHEMBL4016/CHEMBL2093871	Molecule not measured against perfect target	Organism difference/ Protein Complex instead of single Protein	Activities
GRIA2	3kgc	CHEMBL3503				Targets
GRIK1	1vso	CHEMBL2919				Activities
GRIK1	1vso	CHEMBL2919				Targets
HDAC2	3max	CHEMBL1937				Activities
HDAC2	3max	CHEMBL1937				Targets
HDAC8	3f07	CHEMBL3192	CHEMBL2093865	Molecules not measured against perfect target	Protein Familiy instead of single Protein	Activities
HDAC8	3f07	CHEMBL3192				Targets
HIVINT	3nf7	CHEMBL3471		No Activities found		Activities
HIVINT	3nf7	CHEMBL3471	CHEMBL2366505	Sequence matching	Almost identical target	Targets
HIVPR	1xl2	CHEMBL3638326	CHEMBL243		Almost identical target	Activities
HIVPR	1xl2	CHEMBL3638326	CHEMBL5823	Target number is smaller		Targets
HIVRT	3lan	CHEMBL247		No Activities found		Activities
HIVRT	3lan	CHEMBL247				Targets
HMDH	3ccw	CHEMBL402				Activities
HMDH	3ccw	CHEMBL402				Targets
HS90A	1uyg	CHEMBL3880	CHEMBL2095165	Molecules not measured against perfect target	Protein Familiy instead of single Protein	Activities
HS90A	1uyg	CHEMBL3880				Targets
HXK4	3f9m	CHEMBL3820				Activities
HXK4	3f9m	CHEMBL3820				Targets
IGF1R	2oj9	CHEMBL1957				Activities
IGF1R	2oj9	CHEMBL1957				Targets
INHA	4trj	CHEMBL1849				Activities
INHA	4trj	CHEMBL1849				Targets
ITAL	2ica	CHEMBL1803				Activities
ITAL	2ica	CHEMBL1803				Targets
JAK2	3lpb	CHEMBL2971				Activities
JAK2	3lpb	CHEMBL2971				Targets

KIF11	3cjo	CHEMBL4581				Activities
KIF11	3cjo	CHEMBL4581				Targets
KIT	3g0e	CHEMBL1936		No Activities found		Activities
KIT	3g0e	CHEMBL1936				Targets
KITH	2b8t	CHEMBL1075130				Activities
KITH	2b8t	CHEMBL1075130				Targets
KPCB	2i0e	CHEMBL3045		No Activities found		Activities
KPCB	2i0e	CHEMBL3045				Targets
LCK	2of2	CHEMBL258				Activities
LCK	2of2	CHEMBL258				Targets
LKHA4	3chp	CHEMBL4618				Activities
LKHA4	3chp	CHEMBL4618				Targets
MAPK2	3m2w	CHEMBL2208				Activities
MAPK2	3m2w	CHEMBL2208				Targets
MCR	2aa2	CHEMBL1994		No Activities found		Activities
MCR	2aa2	CHEMBL1994				Targets
MET	3lq8	CHEMBL3717				Activities
MET	3lq8	CHEMBL3717				Targets
MK01	2ojg	CHEMBL4040				Activities
MK01	2ojg	CHEMBL4040				Targets
MK10	2zdt	CHEMBL2637	CHEMBL2276	Molecules not measured against perfect target	kinase 1 instead 3	Activities
MK10	2zdt	CHEMBL2637				Targets
MK14	2qd9	CHEMBL260				Activities
MK14	2qd9	CHEMBL260				Targets
MMP13	830c	CHEMBL280				Activities
MMP13	830c	CHEMBL280				Targets
MP2K1	3eqh	CHEMBL3587				Activities
MP2K1	3eqh	CHEMBL3587				Targets
NOS1	1qw6	CHEMBL3048				Activities
NOS1	1qw6	CHEMBL3048				Targets
NRAM	1b9v	CHEMBL3377				Activities
NRAM	1b9v	CHEMBL3377				Targets
PA2GA	1kvo	CHEMBL3474				Activities
PA2GA	1kvo	CHEMBL3474				Targets
PARP1	3l3m	CHEMBL3105				Activities
PARP1	3l3m	CHEMBL3105				Targets
PDE5A	1udt	CHEMBL1827				Activities
PDE5A	1udt	CHEMBL1827				Targets
PGH1	2oyu	CHEMBL2949				Activities
PGH1	2oyu	CHEMBL2949				Targets

PGH2	3ln1	CHEMBL4321		Activities
PGH2	3ln1	CHEMBL4321		Targets
PLK1	2owb	CHEMBL3024		Activities
PLK1	2owb	CHEMBL3024		Targets
PNPH	3bgs	CHEMBL4338		Activities
PNPH	3bgs	CHEMBL4338		Targets
PPARA	2p54	CHEMBL239		Activities
PPARA	2p54	CHEMBL239		Targets
PPARD	2znp	CHEMBL3979		Activities
PPARD	2znp	CHEMBL3979		Targets
PPARG	2gtk	CHEMBL235		Activities
PPARG	2gtk	CHEMBL235		Targets
PRGR	3kba	CHEMBL208		Activities
PRGR	3kba	CHEMBL208		Targets
PTN1	2azr	CHEMBL335	No Activities found	Activities
PTN1	2azr	CHEMBL335		Targets
PUR2	1njs	CHEMBL3972	No Activities found	Activities
PUR2	1njs	CHEMBL3972		Targets
PYGM	1c8k	CHEMBL4696		Activities
PYGM	1c8k	CHEMBL4696		Targets
PYRD	1d3g	CHEMBL1966		Activities
PYRD	1d3g	CHEMBL1966		Targets
RENI	3g6z	CHEMBL286		Activities
RENI	3g6z	CHEMBL286		Targets
ROCK1	2etr	CHEMBL3231		Activities
ROCK1	2etr	CHEMBL3231		Targets
RXRA	1mv9	CHEMBL2061		Activities
RXRA	1mv9	CHEMBL2061		Targets
SAHH	1li4	CHEMBL2664		Activities
SAHH	1li4	CHEMBL2664		Targets
SRC	3el8	CHEMBL3655	No Activities found	Activities
SRC	3el8	CHEMBL3655		Targets
TGFR1	3hmm	CHEMBL4439		Activities
TGFR1	3hmm	CHEMBL4439		Targets
THB	1q4x	CHEMBL1947	No Activities found	Activities
THB	1q4x	CHEMBL1947		Targets
THRB	1ype	CHEMBL204	No Activities found	Activities
THRB	1ype	CHEMBL204		Targets
TRY1	2ayw	CHEMBL3769	No Activities found	Activities
TRY1	2ayw	CHEMBL3769		Targets

TRYB1	2zec	CHEMBL2617		No Activities found		Activities
TRYB1	2zec	CHEMBL2617				Targets
TYSY	1syn	CHEMBL2555	CHEMBL6137	Molecule not measured against perfect target	Organism difference	Activities
TYSY	1syn	CHEMBL2555				Targets
UROK	1sqt	CHEMBL3286				Activities
UROK	1sqt	CHEMBL3286				Targets
VGFR2	2p2i	CHEMBL279				Activities
VGFR2	2p2i	CHEMBL279				Targets
WEE1	3biz	CHEMBL5491				Activities
WEE1	3biz	CHEMBL5491				Targets
XIAP	3hl5	CHEMBL4198				Activities
XIAP	3hl5	CHEMBL4198				Targets

References

(S1) Meyder, A.; Kampen, S.; Sieg, J.; Fährrolfes, R.; Friedrich, N.-O.; Flachsenberg, F.; Rarey, M. StructureProfiler: An All-in-One Tool for 3D Protein Structure Profiling. *Bioinformatics (Oxford, England)* **2019**, *35*, 874–876.

(S2) Meyder, A.; Nittinger, E.; Lange, G.; Klein, R.; Rarey, M. Estimating Electron Density Support for Individual Atoms and Molecular Fragments in X-ray Structures. *Journal of Chemical Information and Modeling* **2017**, *57*, 2437–2447.

(S3) Urbaczek, S.; Kolodzik, A.; Fischer, J. R.; Lippert, T.; Heuser, S.; Groth, I.; Schulz-Gasch, T.; Rarey, M. NAOMI: On the Almost Trivial Task of Reading Molecules from Different File Formats. *Journal of Chemical Information and Modeling* **2011**, *51*, 3199–3207.

(S4) Urbaczek, S.; Kolodzik, A.; Groth, I.; Heuser, S.; Rarey, M. Reading PDB: Perception of Molecules from 3D Atomic Coordinates. *Journal of Chemical Information and Modeling* **2013**, *53*, 76–87.

(S5) Flachsenberg, F.; Ehrt, C.; Gutermuth, T.; Rarey, M. Redocking the PDB. *Journal of Chemical Information and Modeling* **2024**, *64*, 219–237.

(S6) McVey, C. E.; Walsh, M. A.; Dodson, G. G.; Wilson, K. S.; Brannigan, J. A. Crystal structures of penicillin acylase enzyme-substrate complexes: Structural insights into the catalytic mechanism. 2001; `https://doi.org/10.2210/pdb1GM8/pdb`.

(S7) McVey, C. E.; Walsh, M. A.; Dodson, G. G.; Wilson, K. S.; Brannigan, J. A. Crystal Structures of Penicillin Acylase Enzyme-substrate Complexes: Structural Insights into the Catalytic Mechanism. *Journal of Molecular Biology* **2001**, *313*, 139–150.

(S8) Audette, G. F.; Engelmann, R.; Hengstenberg, W.; Deutscher, J.; Hayakawa, K.; Quail, J. W.; Delbaere, L. T. J. CRYSTAL STRUCTURE ANALYSIS OF THE

PHOSPHO-SERINE 46 HPR FROM ENTEROCOCCUS FAECALIS. 2000; https://doi.org/10.2210/pdb1FU0/pdb.

(S9) Audette, G. F.; Engelmann, R.; Hengstenberg, W.; Deutscher, J.; Hayakawa, K.; Quail, J. W.; Delbaere, L. T. J. The 1.9 Å Resolution Structure of Phospho-serine 46 HPr from *Enterococcus faecalis*. *Journal of Molecular Biology* **2000**, *303*, 545–553.

(S10) Zhang, B.; Zhang, Y.; Jing, Z.; Liu, X.; Yang, H.; Liu, H.; Rao, Z.; Jiang, H. The crystal structure of COVID-19 main protease in complex with an inhibitor 11a. 2020; https://doi.org/10.2210/pdb6LZE/pdb.

(S11) Dai, W.; Zhang, B.; Jiang, X.-M.; Su, H.; Li, J.; Zhao, Y.; Xie, X.; Jin, Z.; Peng, J.; Liu, F.; Li, C.; Li, Y.; Bai, F.; Wang, H.; Cheng, X.; Cen, X.; Hu, S.; Yang, X.; Wang, J.; Liu, X.; Xiao, G.; Jiang, H.; Rao, Z.; Zhang, L.-K.; Xu, Y.; Yang, H.; Liu, H. Structure-based design of antiviral drug candidates targeting the SARS-CoV-2 main protease. *Science* **2020**, *368*, 1331–1335.

(S12) Protein Data Bank Contents Guide: Atomic Coordinate Entry Format Description Version 3.30. https://files.wwpdb.org/pub/pdb/doc/format_descriptions/Format_v33_Letter.pdf, accessed on 10/27/2025.

(S13) Ref. S12, pp. 127–128.

(S14) Ref. S12, p. 138.

(S15) Kauer, S.; Schirmer, T. Periplasmic repressor protein YfiR. 2015; https://doi.org/10.2210/pdb4XJY/pdb.

(S16) Safo, M. K.; Moure, C. M.; Burnett, J. C.; Joshi, G. S.; Abraham, D. J. HIGH RESOLUTION CRYSTAL STRUCTURE OF DEOXY HEMOGLOBIN COMPLEXED WITH A POTENT ALLOSTERIC EFFECTOR. 2000; https://doi.org/10.2210/pdb1G9V/pdb.

(S17) Safo, M. K.; Moure, C. M.; Burnett, J. C.; Joshi, G. S.; Abraham, D. J. High-resolution crystal structure of deoxy hemoglobin complexed with a potent allosteric effector. *Protein Science* **2001**, *10*, 951–957.

(S18) Centorrino, F.; Wu, Q.; Ottmann, C. Ternary complex of 14-3-3 sigma, p27pT198 phosphopeptide, and WQ176. 2021; https://doi.org/10.2210/pdb7ORT/pdb.

(S19) Rypniewsky, W. R.; Oestergaard, P.; Noerregaard-Madsen, M.; Dauter, M.; Wilson, K. S. FUSARIUM OXYSPORUM TRYPSIN AT ATOMIC RESOLUTION. 2000; https://doi.org/10.2210/pdb1GDN/pdb.

(S20) Rypniewski, W. R.; Østergaard, P. R.; Nørregaard-Madsen, M.; Dauter, M.; Wilson, K. S. *Fusarium oxysporum* trypsin at atomic resolution at 100 and 283 K: a study of ligand binding. *Acta Crystallographica Section D: Biological Crystallography* **2001**, *57*, 8–19.

(S21) Wright, P. A.; Wilmouth, R. C.; Clifton, I. J.; Schofield, C. J. PORCINE PANCREATIC ELASTASE COMPLEXED WITH ACETYL-VAL-GLU-PRO-ILE-COOH. 2001; https://doi.org/10.2210/pdb1H9L/pdb.

(S22) Wright, P. A.; Wilmouth, R. C.; Clifton, I. J.; Schofield, C. J. Kinetic and crystallographic analysis of complexes formed between elastase and peptides from β-casein. *European Journal of Biochemistry* **2001**, *268*, 2969–2974.

(S23) Correy, G. J.; Fraser, J. S. PanDDA analysis group deposition -- Crystal structure of SARS-CoV-2 NSP3 macrodomain in complex with Z5028367849 - (S) isomer. 2022; https://doi.org/10.2210/pdb5SQM/pdb.

(S24) Gahbauer, S.; Correy, G. J.; Schuller, M.; Ferla, M. P.; Doruk, Y. U.; Rachman, M.; Wu, T.; Diolaiti, M.; Wang, S.; Neitz, R. J.; Fearon, D.; Radchenko, D. S.; Moroz, Y. S.; Irwin, J. J.; Renslo, A. R.; Taylor, J. C.; Gestwicki, J. E.; von Delft, F.;

Ashworth, A.; Ahel, I.; Shoichet, B. K.; Fraser, J. S. Iterative computational design and crystallographic screening identifies potent inhibitors targeting the Nsp3 macrodomain of SARS-CoV-2. *Proceedings of the National Academy of Sciences* **2023**, *120*, e2212931120.

(S25) Ref. S12, pp. 159–160.

(S26) Ghosh, A.; Lima, C. D. Structure of fission yeast RNA polymerase II CTD phosphatase Fcp1-R271A bound to beryllium fluoride. 2015; https://doi.org/10.2210/pdb4XQ0/pdb.

(S27) Schwer, B.; Ghosh, A.; Sanchez, A. M.; Lima, C. D.; Shuman, S. Genetic and structural analysis of the essential fission yeast RNA polymerase II CTD phosphatase Fcp1. *RNA* **2015**, *21*, 1135–1146.

(S28) Beckwith, C. R.; Edwards, M. J.; Clarke, T. Crystal structure of Class 1 cytochrome MtoD from Sideroxydans lithotrophicus ES-1. 2015; https://doi.org/10.2210/pdb4XXL/pdb.

(S29) Beckwith, C. R.; Edwards, M. J.; Lawes, M.; Shi, L.; Butt, J. N.; Richardson, D. J.; Clarke, T. A. Characterization of MtoD from *Sideroxydans lithotrophicus:* a cytochrome c electron shuttle used in lithoautotrophic growth. *Frontiers in Microbiology* **2015**, *6*, 332.

(S30) Leung, A. K. W.; Reynolds, R. C.; Riordan, J. M.; Borhani, D. W. Human Dihydrofolate Reductase Complexed With NADPH and 2,4-Diamino-5-(1-o-carboranylmethyl)-6-methylpyrimidine, A novel boron containing, nonclassical Antifolate. 2005; https://doi.org/10.2210/pdb2C2S/pdb.

(S31) Reynolds, R. C.; Campbell, S. R.; Fairchild, R. G.; Kisliuk, R. L.; Micca, P. L.; Queener, S. F.; Riordan, J. M.; Sedwick, W. D.; Waud, W. R.; Leung, A. K. W.;

Dixon, R. W.; Suling, W. J.; Borhani, D. W. Novel Boron-Containing, Nonclassical Antifolates: Synthesis and Preliminary Biological and Structural Evaluation. *Journal of Medicinal Chemistry* **2007**, *50*, 3283–3289.

(S32) Ref. S12, pp. 187–189.

(S33) Ref. S12, pp. 194–195.

(S34) Gill, M. L.; Strobel, S. A.; Loria, J. P. Crystallization of the Tl+-form of the Oxytricha nova G-quadruplex. 2006; https://doi.org/10.2210/pdb2HBN/pdb.

(S35) Gill, M. L.; Strobel, S. A.; Loria, J. P. Crystallization and characterization of the thallium form of the *Oxytricha nova* G-quadruplex. *Nucleic Acids Research* **2006**, *34*, 4506–4514.

(S36) Stogios, P. J.; Nocek, B.; Xu, X.; Cui, H.; Lowden, M.; Savchenko, A. Crystal structure of a glycosylated endo-beta-1,4-xylanase (glycoside hydrolase family 10/GH10) enzyme from Gloeophyllum trabeum. 2015; https://doi.org/10.2210/pdb4XX6/pdb.

(S37) Ref. S12, pp. 164–165.

(S38) Shen, S. Y.; Yang, Z. Q.; Shao, Z. H. Cryo-EM structure of CB2-G protein complex. 2023; https://doi.org/10.2210/pdb8X3L/pdb.

(S39) Yang, X.; Zhou, P.; Shen, S.; Hu, Q.; Tian, C.; Xia, A.; Wang, Y.; Yang, Z.; Nan, J.; Zhou, Y.; Chen, S.; Tian, X.; Wu, C.; Lin, G.; Zhang, L.; Wang, K.; Zheng, T.; Zou, J.; Yan, W.; Shao, Z.; Yang, S. Entropy drives the ligand recognition in G-protein-coupled receptor subtypes. *Proceedings of the National Academy of Sciences* **2024**, *121*, e2401091121.

(S40) Sonnenburg, E. D.; Bilwes, A.; Hunter, T.; Noel, J. P. Crystal Structure of the Src SH2 Domain Complexed with Peptide (SDpYANFK). 2003; https://doi.org/10.2210/pdb1P13/pdb.

(S41) Sonnenburg, E. D.; Bilwes, A.; Hunter, T.; Noel, J. P. The Structure of the Membrane Distal Phosphatase Domain of RPTPα Reveals Interdomain Flexibility and an SH2 Domain Interaction Region. *Biochemistry* **2003**, *42*, 7904–7914.

(S42) Collier, M. P.; Benesch, J. L. P. Human Hsp27 (HspB1) alpha-crystallin domain in complex with a peptide mimic of its phosphorylatable N-terminal region. 2018; `https://doi.org/10.2210/pdb6GJH/pdb`.

(S43) Collier, M. P.; Alderson, T. R.; de Villiers, C. P.; Nicholls, D.; Gastall, H. Y.; Allison, T. M.; Degiacomi, M. T.; Jiang, H.; Mlynek, G.; Fürst, D. O.; van der Ven, P. F. M.; Djinovic-Carugo, K.; Baldwin, A. J.; Watkins, H.; Gehmlich, K.; Benesch, J. L. P. HspB1 phosphorylation regulates its intramolecular dynamics and mechanosensitive molecular chaperone interaction with filamin C. *Science Advances* **2019**, *5*, eaav8421.

(S44) Ref. S12, pp. 173–174.

(S45) Terzyan, S.; Hanigan, M. Crystal Structure of human GGT1 in complex with DON. 2017; `https://doi.org/10.2210/pdb5V4Q/pdb`.

(S46) Terzyan, S. S.; Cook, P. F.; Heroux, A.; Hanigan, M. H. Structure of 6-diazo-5-oxo-norleucine-bound human gamma-glutamyl transpeptidase 1, a novel mechanism of inactivation. *Protein Science* **2017**, *26*, 1196–1205.

(S47) Wagner, T.; Lemaire, O. N.; Engilberge, S. Crystal structure of the ethyl-coenzyme M reductase from Candidatus Ethanoperedens thermophilum gassed with xenon. 2020; `https://doi.org/10.2210/pdb7B2C/pdb`.

(S48) Hahn, C. J.; Lemaire, O. N.; Kahnt, J.; Engilberge, S.; Wegener, G.; Wagner, T. Crystal structure of a key enzyme for anaerobic ethane activation. *Science* **2021**, *373*, 118–121.

(S49) Mathews, I.; Tran, K.; Boxer, S. G. Crystal structure of R. sphaeroides Photosynthetic Reaction Center variant Y(M210)2-cyanophenylalanine. 2024; `https://doi.org/10.2210/pdb8VTJ/pdb`.

(S50) Tran, K. N.; Faries, K. M.; Magdaong, N. C. M.; Mathews, I. I.; Weaver, J. B.; Kirsh, J. M.; Holten, D.; Kirmaier, C.; Boxer, S. G. Application of Amber Suppression To Study the Role of Tyr M210 in Electron Transfer in *Rhodobacter sphaeroides* Photosynthetic Reaction Centers. *The Journal of Physical Chemistry B* **2025**, *129*, 3317–3333.

(S51) Sainz-Polo, M. A.; Sanz-Aparicio, J. Structure of the CBM22-2 xylan-binding domain from Paenibacillus barcinonensis Xyn10C in complex with xylotriose. 2015; `https://doi.org/10.2210/pdb4XUQ/pdb`.

(S52) Sainz-Polo, M. A.; González, B.; Menéndez, M.; Pastor, F. I. J.; Sanz-Aparicio, J. Exploring Multimodularity in Plant Cell Wall Deconstruction: STRUCTURAL AND FUNCTIONAL ANALYSIS OF Xyn10C CONTAINING THE CBM22-1–CBM22-2 TANDEM. *Journal of Biological Chemistry* **2015**, *290*, 17116–17130.

(S53) Carrizo, M. E.; Capaldi, S.; Perduca, M.; Irazoqui, F. J.; Nores, G. A.; Monaco, H. L. Crystal structure of the tetragonal form of the common edible mushroom (Agaricus bisporus) lectin in complex with T-antigen and N-acetylglucosamine. 2004; `https://doi.org/10.2210/pdb1Y2X/pdb`.

(S54) Carrizo, M. E.; Capaldi, S.; Perduca, M.; Irazoqui, F. J.; Nores, G. A.; Monaco, H. L. The Antineoplastic Lectin of the Common Edible Mushroom (*Agaricus bisporus*) Has Two Binding Sites, Each Specific for a Different Configuration at a Single Epimeric Hydroxyl. *Journal of Biological Chemistry* **2005**, *280*, 10614–10623.

(S55) Ref. S12, pp. 164–165.

(S56) Muckelbauer, J. K. Crystal structure of bruton agammaglobulinemia tyrosine kinase complexed with BMS-824171 AKA 6-[(3R)-3-(4-tert-bu tylbenzamido)piperidin-1-yl]-2-{[4-(morpholine-4-carbonyl) phenyl]amino}pyridine-3-carboxamide. 2015; https://doi.org/10.2210/pdb5BQ0/pdb.

(S57) Liu, Q.; Batt, D. G.; Lippy, J. S.; Surti, N.; Tebben, A. J.; Muckelbauer, J. K.; Chen, L.; An, Y.; Chang, C.; Pokross, M.; Yang, Z.; Wang, H.; Burke, J. R.; Carter, P. H.; Tino, J. A. Design and synthesis of carbazole carboxamides as promising inhibitors of Bruton's tyrosine kinase (BTK) and Janus kinase 2 (JAK2). *Bioorganic & Medicinal Chemistry Letters* **2015**, *25*, 4265–4269.

(S58) Zhao, F.; Fan, R. Y.; Guan, Z. Y.; Guo, L.; Yin, P. An auto-activation mechanism of plant non-specific phospholipase C. 2022; https://doi.org/10.2210/pdb8HAW/pdb.

(S59) Fan, R.; Zhao, F.; Gong, Z.; Chen, Y.; Yang, B.; Zhou, C.; Zhang, J.; Du, Z.; Wang, X.; Yin, P.; Guo, L.; Liu, Z. Insights into the mechanism of phospholipid hydrolysis by plant non-specific phospholipase C. *Nature Communications* **2023**, *14*, 194.

(S60) Ref. S12, pp. 159–160.

(S61) McCoy, J. G.; Wesenberg, G. E.; Phillips Jr., G. N.; Bitto, E.; Bingman, C. A.; Center for Euraryotic Structural Genomics (CESG)., Crystal Structure of a Putative UDP-glucose Pyrophosphorylase from Arabidopsis Thaliana with Bound UDP-glucose. 2006; https://doi.org/10.2210/pdb2ICY/pdb.

(S62) McCoy, J. G.; Bitto, E.; Bingman, C. A.; Wesenberg, G. E.; Bannen, R. M.; Kondrashov, D. A.; Phillips Jr, G. N. Structure and Dynamics of UDP–Glucose Pyrophosphorylase from *Arabidopsis thaliana* with Bound UDP–Glucose and UTP. *Journal of Molecular Biology* **2007**, *366*, 830–841.

(S63) RDKit: Open-source Cheminformatics. http://www.rdkit.org.

(S64) Koua, F.; Guenther, S.; Reinke, P.; Oberthuer, D.; Yefanov, O.; Gelisio, L.; Ginn, H.; Lieske, J.; Ewert, W.; Domaracky, M.; Brehm, W.; Rahmani Mashour, A.; White, T.; Knoska, J.; Pena Esperanza, G.; Tolstikova, A.; Groessler, M.; Fischer, P.; Hennicke, V.; Fleckenstein, H.; Trost, F.; Galchenkova, M.; Gevorkov, Y.; Li, C.; Awel, S.; Paulraj, L.; Ullah, N.; Falke, S.; Alves Franca, B.; Schwinzer, M.; Brognaro, H.; Werner, N.; Perbandt, M.; Tidow, H.; Seychell, B.; Beck, T.; Meier, S.; Doyle, J.; Giseler, H.; Melo, D.; Dunkel, I.; Lane, T.; Peck, A.; Saouane, S.; Hakanpaeae, J.; Meyer, J.; Noei, H.; Gribbon, P.; Ellinger, B.; Kuzikov, M.; Wolf, M.; Zhang, L.; Ehrt, C.; Pletzer-Zelgert, J.; Wollenhaupt, J.; Feiler, C.; Weiss, M.; Schulz, E.; Mehrabi, P.; Norton-Baker, B.; Schmidt, C.; Lorenzen, K.; Schubert, R.; Han, H.; Chari, A.; Fernandez Garcia, Y.; Turk, D.; Hilgenfeld, R.; Rarey, M.; Zaliani, A.; Chapman, H.; Pearson, A.; Betzel, C.; Meents, A. Structure of SARS-CoV-2 Main Protease Bound to Ifenprodil: 7aqi. 2020; https://www.wwpdb.org/pdb?id=pdb_00007aqi.

(S65) Günther, S.; Reinke, P. Y. A.; Fernández-García, Y.; Lieske, J.; Lane, T. J.; Ginn, H. M.; Koua, F. H. M.; Ehrt, C.; Ewert, W.; Oberthuer, D.; Yefanov, O.; Meier, S.; Lorenzen, K.; Krichel, B.; Kopicki, J.-D.; Gelisio, L.; Brehm, W.; Dunkel, I.; Seychell, B.; Gieseler, H.; Norton-Baker, B.; Escudero-Pérez, B.; Domaracky, M.; Saouane, S.; Tolstikova, A.; White, T. A.; Hänle, A.; Groessler, M.; Fleckenstein, H.; Trost, F.; Galchenkova, M.; Gevorkov, Y.; Li, C.; Awel, S.; Peck, A.; Barthelmess, M.; Schlünzen, F.; Lourdu Xavier, P.; Werner, N.; Andaleeb, H.; Ullah, N.; Falke, S.; Srinivasan, V.; França, B. A.; Schwinzer, M.; Brognaro, H.; Rogers, C.; Melo, D.; Zaitseva-Doyle, J. J.; Knoska, J.; Peña-Murillo, G. E.; Mashhour, A. R.; Hennicke, V.; Fischer, P.; Hakanpää, J.; Meyer, J.; Gribbon, P.; Ellinger, B.; Kuzikov, M.; Wolf, M.; Beccari, A. R.; Bourenkov, G.; Von Stetten, D.; Pompidor, G.; Bento, I.; Panneerselvam, S.; Karpics, I.; Schneider, T. R.; Garcia-Alai, M. M.; Niebling, S.; Günther, C.; Schmidt, C.; Schubert, R.; Han, H.; Boger, J.; Monteiro, D. C. F.; Zhang, L.;

Sun, X.; Pletzer-Zelgert, J.; Wollenhaupt, J.; Feiler, C. G.; Weiss, M. S.; Schulz, E.-C.; Mehrabi, P.; Karničar, K.; Usenik, A.; Loboda, J.; Tidow, H.; Chari, A.; Hilgenfeld, R.; Uetrecht, C.; Cox, R.; Zaliani, A.; Beck, T.; Rarey, M.; Günther, S.; Turk, D.; Hinrichs, W.; Chapman, H. N.; Pearson, A. R.; Betzel, C.; Meents, A. X-Ray Screening Identifies Active Site and Allosteric Inhibitors of SARS-CoV-2 Main Protease. *Science* **2021**, *372*, 642–646.

(S66) Correy, G.; Young, I.; Thompson, M.; Fraser, J. PanDDA Analysis Group Deposition – Crystal Structure of SARS-CoV-2 NSP3 Macrodomain in Complex with ZINC000008652361: 5rt2. 2020; `https://www.wwpdb.org/pdb?id=pdb_00005rt2`.

(S67) Schuller, M.; Correy, G. J.; Gahbauer, S.; Fearon, D.; Wu, T.; Díaz, R. E.; Young, I. D.; Carvalho Martins, L.; Smith, D. H.; Schulze-Gahmen, U.; Owens, T. W.; Deshpande, I.; Merz, G. E.; Thwin, A. C.; Biel, J. T.; Peters, J. K.; Moritz, M.; Herrera, N.; Kratochvil, H. T.; QCRG Structural Biology Consortium,; Aimon, A.; Bennett, J. M.; Brandao Neto, J.; Cohen, A. E.; Dias, A.; Douangamath, A.; Dunnett, L.; Fedorov, O.; Ferla, M. P.; Fuchs, M. R.; Gorrie-Stone, T. J.; Holton, J. M.; Johnson, M. G.; Krojer, T.; Meigs, G.; Powell, A. J.; Rack, J. G. M.; Rangel, V. L.; Russi, S.; Skyner, R. E.; Smith, C. A.; Soares, A. S.; Wierman, J. L.; Zhu, K.; O'Brien, P.; Jura, N.; Ashworth, A.; Irwin, J. J.; Thompson, M. C.; Gestwicki, J. E.; Von Delft, F.; Shoichet, B. K.; Fraser, J. S.; Ahel, I. Fragment Binding to the Nsp3 Macrodomain of SARS-CoV-2 Identified through Crystallographic Screening and Computational Docking. *Science Advances* **2021**, *7*, eabf8711.

(S68) Wojdyr, M. GEMMI: A Library for Structural Biology. *Journal of Open Source Software* **2022**, *7*, 4200.

(S69) deSolms, S. J.; Ciccarone, T.; MacTough, S.; Shaw, A. W.; Buser, C.; Ellis-Hutchings, M.; Fernandes, C.; Hamilton, K.; Huber, H.; Kohl, N.; Lobell, R.; Robinson, R.; Tsou, N.; Walsh, E.; Graham, S.; Beese, L.; Taylor, J. Co-Crystal Structure

Of Human Farnesyltransferase With Farnesyldiphosphate and Inhibitor Compound 33a. 2002; https://doi.org/10.2210/pdb1MZC/pdb.

(S70) deSolms, S. J.; Ciccarone, T. M.; MacTough, S. C.; Shaw, A. W.; Buser, C. A.; Ellis-Hutchings, M.; Fernandes, C.; Hamilton, K. A.; Huber, H. E.; Kohl, N. E.; Lobell, R. B.; Robinson, R. G.; Tsou, N. N.; Walsh, E. S.; Graham, S. L.; Beese, L. S.; Taylor, J. S. Dual Protein Farnesyltransferase-Geranylgeranyltransferase-I Inhibitors as Potential Cancer Chemotherapeutic Agents. *Journal of Medicinal Chemistry* **2003**, *46*, 2973–2984.

(S71) Pavlicek, J.; Ptacek, J.; Cerny, J.; Byun, Y.; Skultetyova, L.; Pomper, M.; Lubkowski, J.; Barinka, C. X-Ray Structure of of Human Glutamate Carboxypeptidase II (GCPII) in a Complex with CCIBzL, a Urea-Based Inhibitor N~2~-[(1-Carboxycyclopropyl)Carbamoyl]-N~6~-(4-Iodobenzoyl)-L-lysine: 4oc0. 2014; https://www.wwpdb.org/pdb?id=pdb_00004oc0.

(S72) Pavlicek, J.; Ptacek, J.; Cerny, J.; Byun, Y.; Skultetyova, L.; Pomper, M. G.; Lubkowski, J.; Barinka, C. Structural Characterization of P1′-Diversified Urea-Based Inhibitors of Glutamate Carboxypeptidase II. *Bioorganic & Medicinal Chemistry Letters* **2014**, *24*, 2340–2345.

(S73) Fährrolfes, R.; Bietz, S.; Flachsenberg, F.; Meyder, A.; Nittinger, E.; Otto, T.; Volkamer, A.; Rarey, M. Proteins Plus: A Web Portal for Structure Analysis of Macromolecules. *Nucleic Acids Research* **2017**, *45*, W337–W343.

(S74) Schöning-Stierand, K.; Diedrich, K.; Fährrolfes, R.; Flachsenberg, F.; Meyder, A.; Nittinger, E.; Steinegger, R.; Rarey, M. Proteins Plus: Interactive Analysis of Protein–Ligand Binding Interfaces. *Nucleic Acids Research* **2020**, *48*, W48–W53.

(S75) Schöning-Stierand, K.; Diedrich, K.; Ehrt, C.; Flachsenberg, F.; Graef, J.; Sieg, J.; Penner, P.; Poppinga, M.; Ungethüm, A.; Rarey, M. Proteins Plus: A Comprehensive

Collection of Web-Based Molecular Modeling Tools. *Nucleic Acids Research* **2022**, *50*, W611–W615.

(S76) Mysinger, M. M.; Carchia, M.; Irwin, J. J.; Shoichet, B. K. Directory of Useful Decoys, Enhanced (DUD-E): Better Ligands and Decoys for Better Benchmarking. *Journal of Medicinal Chemistry* **2012**, *55*, 6582–6594.

(S77) Suino-Powell, K.; Xu, Y.; Zhang, C.; Tao, Y.-g.; Tolbert, W. D.; Simons, S. S.; Xu, H. E. Doubling the Size of the Glucocorticoid Receptor Ligand Binding Pocket by Deacylcortivazol. *Molecular and Cellular Biology* **2008**, *28*, 1915–1923.

(S78) Xu, H. Doubling the Size of the Glucocorticoid Receptor Ligand Binding Pocket by Deacylcortivazol: 3bqd. 2008; `https://www.wwpdb.org/pdb?id=pdb_00003bqd`.

(S79) Sobolevsky, A.; Rosconi, M.; Gouaux, E. Isolated Ligand Binding Domain Dimer of GluA2 Ionotropic Glutamate Receptor in Complex with Glutamate, LY 404187 and ZK 200775: 3kgc. 2009; `https://www.wwpdb.org/pdb?id=pdb_00003kgc`.

(S80) Hast, M. A.; Fletcher, S.; Cummings, C. G.; Pusateri, E. E.; Blaskovich, M. A.; Rivas, K.; Gelb, M. H.; Van Voorhis, W. C.; Sebti, S. M.; Hamilton, A. D.; Beese, L. S. Structural Basis for Binding and Selectivity of Antimalarial and Anticancer Ethylenediamine Inhibitors to Protein Farnesyltransferase. *Chemistry & Biology* **2009**, *16*, 181–192.

(S81) Hast, M.; Beese, L. Protein Farnesyltransferase Complexed with Bisubstrate Ethylenediamine Scaffold Inhibitor 5. 2009; `https://doi.org/10.2210/pdb1E37/pdb`.

(S82) Lippa, B.; Pan, G.; Corbett, M.; Li, C.; Kauffman, G. S.; Pandit, J.; Robinson, S.; Wei, L.; Kozina, E.; Marr, E. S.; Borzillo, G.; Knauth, E.; Barbacci-Tobin, E. G.; Vincent, P.; Troutman, M.; Baker, D.; Rajamohan, F.; Kakar, S.; Clark, T.; Morris, J. Synthesis and Structure Based Optimization of Novel Akt Inhibitors. *Bioorganic & Medicinal Chemistry Letters* **2008**, *18*, 3359–3363.

(S83) Pandit, J. Crystal Structure of Akt-1 Complexed with Substrate Peptide and Inhibitor: 3cqw. 2008; https://www.wwpdb.org/pdb?id=pdb_00003cqw.

(S84) Boettcher, J.; Specker, E.; Heine, A.; Klebe, G. HIV-1 Protease in Complex with Pyrrolidinmethanamine: 1xl2. 2005; https://www.wwpdb.org/pdb?id=pdb_00001xl2.

(S85) Specker, E.; Böttcher, J.; Lilie, H.; Heine, A.; Schoop, A.; Müller, G.; Griebenow, N.; Klebe, G. An Old Target Revisited: Two New Privileged Skeletons and an Unexpected Binding Mode For HIV-Protease Inhibitors. *Angewandte Chemie International Edition* **2005**, *44*, 3140–3144.

(S86) Peat, T.; Newman, J.; Deadman, J.; Rhodes, D. Structural Basis for a New Mechanism of Inhibition of HIV Integrase Identified by Fragment Screening and Structure Based Design: 3nf7. 2011; https://www.wwpdb.org/pdb?id=pdb_00003nf7.

(S87) Rhodes, D. I.; Peat, T. S.; Vandegraaff, N.; Jeevarajah, D.; Le, G.; Jones, E. D.; Smith, J. A.; Coates, J. A.; Winfeld, L. J.; Thienthong, N.; Newman, J.; Lucent, D.; Ryan, J. H.; Savage, G. P.; Francis, C. L.; Deadman, J. J. Structural Basis for a New Mechanism of Inhibition of H I V-1 Integrase Identified by Fragment Screening and Structure-Based Design. *Antiviral Chemistry and Chemotherapy* **2011**, *21*, 155–168.
